# An IgA1-lambda-type monoclonal immunoglobulin deposition disease associated with membranous features in a patient with IgG4-related kidney disease: a case report

**DOI:** 10.1186/s12882-018-1133-9

**Published:** 2018-11-20

**Authors:** Atsushi Kitazawa, Ryo Koda, Atsunori Yoshino, Yoshihiko Ueda, Tetsuro Takeda

**Affiliations:** 1 Department of Nephrology, Dokkyo Medical University Saitama Medical Center, 2-1-50 Minamikoshigaya, Koshigaya, Saitama, 343-8555 Japan; 2 Department of Pathology, Dokkyo Medical University Saitama Medical Center, 2-1-50 Minamikoshigaya, Koshigaya, Saitama, 343-8555 Japan

**Keywords:** IgG4-related disease, Monoclonal immunoglobulin deposition disease, Membranous nephropathy, Tubulointerstitial nephritis

## Abstract

**Background:**

IgG4-related disease (IgG4-RD) is a newly recognized fibroinflammatory condition. The kidney is one of the organs commonly affected by IgG4-RD. Tubulointerstitial nephritis (TIN) is the main feature, and membranous nephropathy (MN) has also been described frequently. In MN, polyclonal immunoglobulins and complements are deposited in granular form along the glomerular basement membranes (GBMs). Unusual cases of monoclonal immunoglobulin deposition disease (MIDD) associated with membranous features have been reported. MIDD is morphologically similar to MN but contains immunoglobulins considered to be derived from single B-cell clone.

**Case presentation:**

We describe a 65-year-old man who was referred to our hospital because of hyperproteinaemia, eosinophilia, anaemia, and proteinuria. A renal biopsy demonstrated infiltration of plasma cells and eosinophils in the interstitium, and the ratio of IgG4-positive plasma cells to IgG-positive plasma cells was 55%. The patient was diagnosed as having IgG4-related TIN. Periodic acid methenamine silver staining under light microscopy revealed a bubbling appearance and spike formation in the GBM. On immunofluorescence, the expression of IgG and complements was negative; however, IgA was positively expressed in a granular pattern along the GBM. An IgA subclass analysis revealed a significant deposition of IgA1-lambda (IgA1-λ). Electron microscopy revealed irregular and small non-organized and non-Randall-type granular electron-dense deposits in the GBM that were shaped like snow leopard spots.

**Conclusions:**

After corticosteroid therapy was initiated, the patient’s eosinophilia remarkably improved and his serum creatinine, IgG, and IgG4 levels decreased to within the normal ranges. However, massive proteinuria persisted. To our knowledge, this is the first reported case of IgG4-related TIN associated with IgA1-λ-type MIDD with membranous features.

## Background

Membranous nephropathy (MN) is defined as glomerulonephritis with a bubbling appearance and formation of spikes in the glomerular basement membrane (GBM) on light microscopy. On immunofluorescence, polyclonal immunoglobulins and complements are deposited in granular form along the glomerular basement membranes (GBMs). Only a few cases of non-organized and non-Randall-type monoclonal immunoglobulin deposition disease (MIDD) associated with membranous features have been reported in the literature [1, 2]. MIDD is similar to MN; however, on immunofluorescence, MIDD contains immunoglobulins restricted to a single immunoglobulin class, a single immunoglobulin subclass, and a single light chain, consistent with monoclonal proteins [1, 2].

On the other hand, IgG4-related disease (IgG4-RD) is recognized as a new chronic inflammatory disease characterized by elevated serum IgG4 levels, mass or tissue infiltration rich in IgG4-positive plasma cells, and storiform fibrosis [3].

The kidney is one of the organs commonly affected by IgG4-RD, and tubulointerstitial nephritis (TIN) with infiltration of numerous IgG4-positive plasma cells is the most common type of kidney lesion, and MN has been occasionally accompanied [4–6].

Here, we describe a patient with IgG4-related kidney disease who developed massive proteinuria due to membranous features associated with the deposition of IgA1-lambda (IgA1-λ) along the glomerular capillary walls.

## Case presentation

A 65-year-old man was referred to our hospital because of hyperproteinaemia, eosinophilia, anaemia, and proteinuria after a 2-week history of slight fever, fatigue, and malaise.

On admission, his mental status was normal, body temperature was 36.5 °C, pulse was 73 bpm and regular, and blood pressure was 118/75 mmHg. A physical examination revealed eruption and oedema in his lower extremities; however, no abnormal signs were observed in the lungs, heart, or abdomen. His lymph node and thyroid gland were not swollen. The laboratory findings on admission are summarized in Table 1. In brief, the eosinophil count was markedly increased (50%). The IgG and IgG4 levels were markedly increased (6380 and 2430 mg/dL, respectively). Urinalysis revealed massive proteinuria (3.5 g/day) with haematuria (5–10 per high-power field), and the β2-microglobulin level was 2863 ng/mL. Chest radiography revealed ground-glass opacities in the lower lung field. Chest computed tomography (CT) revealed bronchial wall thickening and ground-glass opacities in the right middle and lower lobes of the lung. Abdominal CT revealed bilateral renal enlargement.

**Table 1:** Laboratory findings on admission

*Urinalysis*		*Blood chemistry*		*Immuno-serological findings*	
Pro.	(3+)	TP	10.3 g/dL	CRP	1.6 mg/dL
Glu.	(−)	Alb	2.2 g/dL	IgG	6380 mg/dL
Ket.	(−)	T-bil	0.8 mg/dL	IgA	90 mg/dL
Occult blood	(2+)	AST	21 IU/L	IgM	42 mg/dL
Sed.		ALT	11 IU/L	IgE	1480 IU/mL
RBC	10–19/HPF	LDH	191 IU/L	IgG4	2430 mg/dL
WBC	5–9/HPF	ALP	265 IU/L	C3	56 mg/dL
Cast	(+)	γ-GTP	24 IU/L	C4	4.5 mg/dL
Bence-Jones protein	(−)	BUN	27 mg/dL	Anti-nuclear-Ab	(−)
*CBC*		Cr	1.4 mg/dL	MPO-ANCA	(−)
WBC	9300 /μL	Na	133 mEq/L	PR3-ANCA	(−)
(polys)	35%	K	4.7 mEq/L	Cryoglobulin	(−)
(lym)	13%	Cl	105 mEq/L	IEP	(−)
(mono)	2%	Ca	8.1 mg/dL	FLC κ/λ retio	1.35
(eosino)	50%	IP	4.3 mg/dL	HBs-Ag	(−)
RBC	314 × 104/μL	UA	7.1 mg/dL	HCV-Ab	(−)
Hb	9.4 g/dl	CK	28 IU/L	T-spot	(−)
Ht	28.2%	TSH	4.23 μU/ml		
Plt	25.4 × 104/μL	FT4	0.99 ng/dl		

**Fig. 1:**
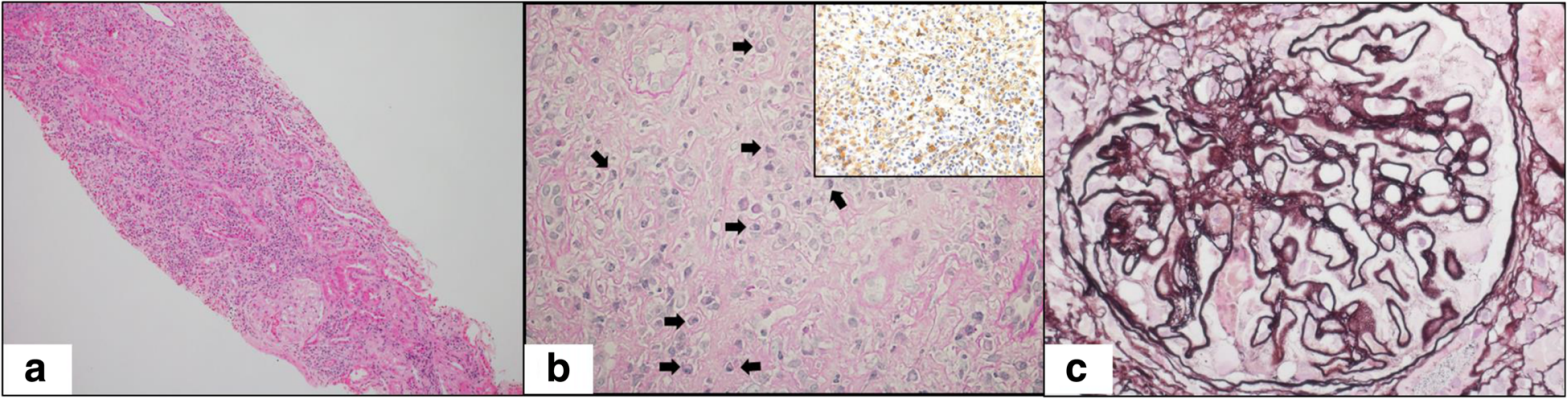
Light microscopy findings of the renal biopsy specimen. **a** Interstitium showing extensive plasma cell infiltration, partial accumulation of eosinophils, and lymphocytes (haematoxylin-eosin staining, original magnification × 100). **b** Interstitium showing plasma cell infiltration (arrow) and storiform fibrosis with tubule atrophy (periodic acid-Schiff staining, original magnification × 400). Marked increase in IgG4-positive plasma cells was seen in the infiltrate (immunofluorescence staining for IgG4, original magnification × 400). **c** The glomeruli showing spike formation and bubbling on the glomerular capillary walls (periodic acid methenamine silver-Masson trichrome, original magnification × 1000)

A renal biopsy was performed. Light microscopy revealed 3 global scleroses and no crescent within the 9 glomeruli. In the interstitium, severe infiltration of plasma cells and eosinophils, with storiform fibrosis and infiltration of numerous IgG4-positive plasma cells (IgG4-/IgG-positive plasma cell ratio > 50%) were observed (Fig. 1a, b). In the functioning glomeruli, the GBM had a bubbling appearance with spikes but without significant mesangial cell or matrix proliferation (Fig. 1c). Direct fast scarlet staining was negative.

**Fig. 2:**
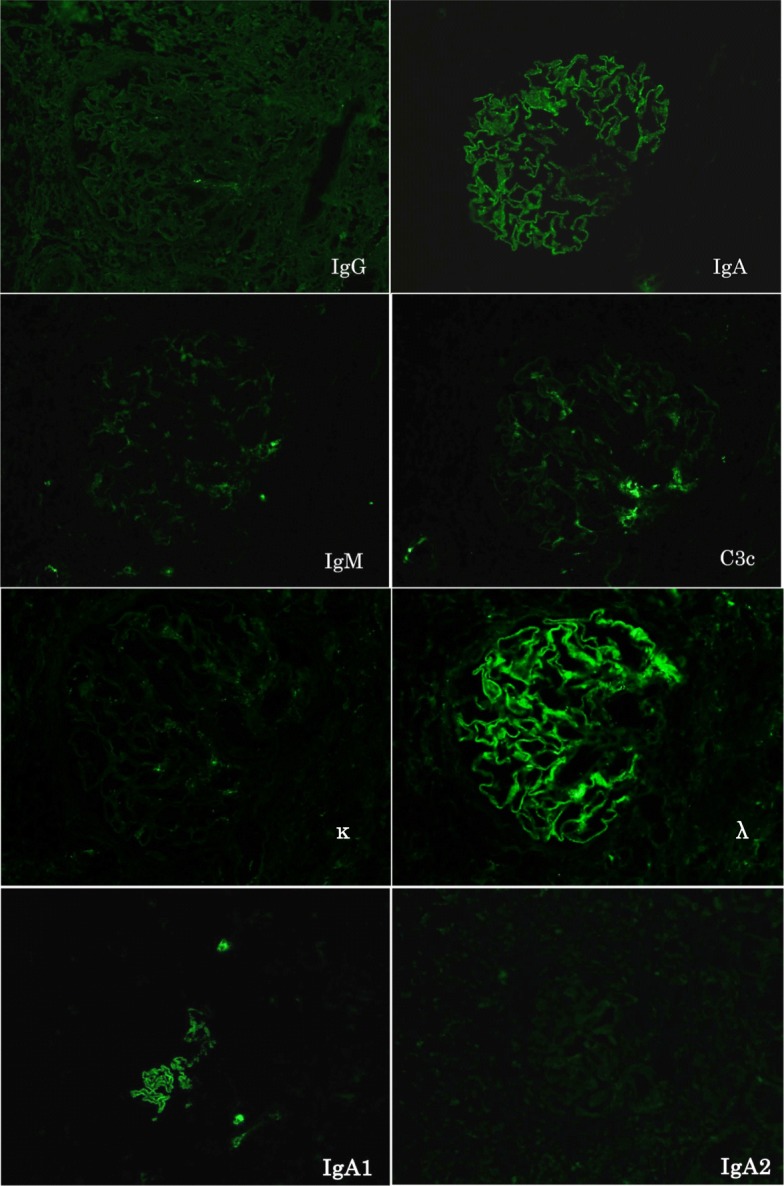
Immunofluorescence microscopy image for the IgG heavy chain; κ-light chain; λ-light chain; C3; and IgG heavy-chain subclasses of IgG1, IgG2, IgG3, IgG4, IgA1, and IgA2. Granular staining of IgA heavy chain dominant for IgA1 and λ-light chain along the glomerular capillary walls is shown (original magnification × 400)

On immunofluorescence, the expression of IgG and complements was negative; however, IgA was positively expressed in a granular pattern along the GBM. An IgA subclass analysis revealed significant monoclonal deposition of IgA1-λ (Fig. 2). We cut the frozen sections of renal biopsy specimens several times for other purposes such as immunostaining. Therefore, the last cut section was used for IgA subclass analysis, and there were few residual tissues and only an obliquely cut segment of the glomerulus remained. For this reason, we believe that it seemed that only a segment of the glomerulus stained with IgA1. Immunofluorescence staining for antibodies to M-type phospholipase A2 receptor (PLA2R) was negative (data not shown).

**Fig. 3:**
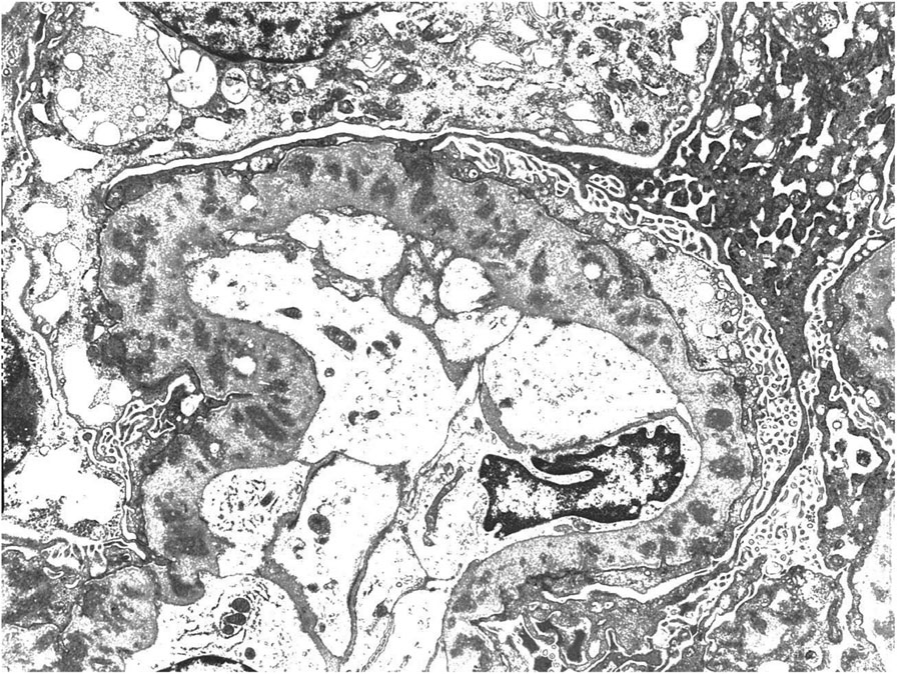
Electron microscopy image showing granular electron-dense deposits without organized structures in the basement membrane

Electron microscopy revealed GBM thickening, widespread podocyte effacement, and irregular and small non-organized and non-Randall-type granular electron-dense deposits in the GBM (Fig. 3) that were shaped like snow leopard spot-like pattern. On the basis of these findings, the diagnosis was IgA1-lambda-type non-Randall monoclonal immunoglobulin deposition disease associated with membranous features in a patient with IgG4-related TIN, according to the criteria for IgG4-RD [1]. Corticosteroid therapy was initiated with 3 days of 500 mg intravenous methyl-prednisolone, followed by 40 mg/day prednisolone. After the treatment, the oedema, eruption, and eosinophilia remarkably improved immediately. The serum creatinine level decreased to 1.0 mg/dL, and IgG and IgG4 levels decreased to within their normal ranges. However, massive proteinuria persisted.

## Discussion and conclusions

IgG4-RD is a recently recognized systemic immune-mediated disease, typically characterized by mass-forming fibroinflammatory lesions. IgG4-RD is well recognized in the form of TIN (IgG4-related TIN), which may present marked infiltration of lymphocytes and IgG4-positive plasma cells into the renal interstitium with interstitial fibrosis and minor glomerular lesions [7, 8].

However, several case reports have described IgG4-RD showing glomerular changes, of which MN is the most common. MN is present in approximately 7% of patients with IgG4-related TIN [9].

In MN associated with IgG4-RD, positive deposition of IgG4 without light-chain monoclonality in the GBM has been observed, either predominantly or together with other IgG subclasses [5, 9–12]. MN secondary to IgG4-RD is termed ‘IgG4-related MN’ [3], although primary MN is recognized to also have a dominant positive deposition of IgG4 in the GBM. Thus, immunostaining for IgG subclasses would likely not distinguish between primary MN and IgG4-related MN.

Alexander et al. reported that the glomerular staining tests for anti-PLA2R in 8 biopsies of MN in the setting of IgG4-RD were all negative. Thus, they argued that IgG4-related MN is a secondary MN [5]. In our case, glomerular staining for anti-PLA2R in biopsy was also negative.

In the present case, although serum IgG and IgG4 levels were elevated beyond the physiological level, routine techniques failed to detect an underlying B-cell disorder, with negative serum and urine protein immunoelectrophoresis and a normal free light chain (FLC) ratio. In Brief, the present case was compatible with IgG4-related kidney disease, however bone marrow examinations were not performed, so the possibility of hematological disorders could not be completely denied.

According to the systematic review of 37 cases of IgG4-related kidney disease [6], the serum IgG and IgG4 levels were elevated to 1920–8194 mg/dL and 221-4630 mg/dL, respectively. Most cases of IgG4-related kidney disease show no elevation of IgM and IgA. However, IgM and IgA levels usually increase in TIN associated with autoimmune and inflammatory diseases. This is an important differential point. In the present case, the IgG level was 6380 mg/dL, IgG4 level was 2430 mg/dL, and IgA level was not elevated.

We did not examine viral antibodies such as Epstein-Barr virus and human herpesvirus-8; however, liver function was normal and atypical lymphocytes were not observed. Splenomegaly was not observed and the lymph nodes were not swollen; thus, it is unlikely that the patient had those viral infections.

CT scan did not reveal splenomegaly and lymphadenopathy. Moreover, there were no abnormal findings in the pancreas, salivary gland, and parotid.

IgG4-RD can be complicated with Sjogren syndrome or multicentric Castleman disease (MCD), and some cases are difficult to differentially diagnose.

The underlying premise is that the histological features (dense lymphoplasmacytic infiltrate and fibrosis, arranged at least focally in a storiform pattern) associated with IgG4-RD are highly specific when viewed in conjunction with IgG4 staining [13].

The presence of an atopic history is characteristic of IgG4-RD, whereas intense inflammation and polyclonal hyperimmunoglobulinaemia are characteristics of MCD. Moreover, IgA and C-reactive protein (CRP) levels are significantly higher in MCD than in IgG4-RD [14].

In the present case, the IgA level was not elevated and the CRP level was 1.6 mg/dL. Moreover, also consistent with IgG4-RD, the IgG, IgG4, and complement levels promptly returned to the normal ranges after corticosteroid use.

In 2004, Nasr et al. first described proliferative glomerulonephritis with monoclonal IgG deposits (PGNMID), a newly characterized form of glomerulonephritis related to monoclonal IgG deposition that could not be assigned to any of the established categories of glomerular diseases. The glomerular deposits appeared primarily granular, resembling ordinary immune complex deposits but contained immunoglobulins considered to be derived from single B cell clone. They classified the disease, according to the pattern of proliferative changes on light microscopy, as membranous proliferative glomerulonephritis, endocapillary proliferative glomerulonephritis, mesangial proliferative glomerulonephritis, and membranous glomerulonephritis [1].

Recently, Komatsuda et al. reported 3 patients with PGNMID with membranous features (2 IgG3-κ and 1 IgG1-κ) [2]. The condition was named ‘non-Randall MIDD’ because PGNMID with membranous features are poor in proliferative changes.

Eighteen cases of non-Randall MIDD with membranous features have been reported (Table 2), and IgG subclass analysis along the GBM mostly showed IgG1 or IgG3. The presence of monoclonal gammopathy in the sera is rare.

**Table 2:** Summary of Reported Cases of Non Randal Monoclonal Immunoglobulin Deposition Disease with Membranous Features

		First author	age	sex	IEP	IF	EM	Aetiology	treatment	outcome
1	2003	Touchard [20]	48	M	no	IgG3λ	subepi, subendo, mes	Idiopathic	PSL, Melphalan	remission
2	2003	Evans [21]	81	F	n/a	IgG1κ	subepi	ML (B cell)	PSL, CY, Chlorambucil	dead
3	2004	Nasr [1]	63	F	IgGλ	IgG1κ	subepi, subendo, mes	Idiopathic		n/a
4			42	M	no	IgG1κ	subepi, subendo	Idiopathic	PSL	decreased proteinuria
5	2008	Komatsuda [2]	44	M	no	IgG3κ	subepi	Idiopathic	mPSL, PSL	decreased proteinuria
6			24	M	no	IgG3κ	subepi, subendo, mes	Idiopathic	PSL	decreased proteinuria
7	2010	Miura [15]	61	M	no	IgA1λ	subepi	HCV, cancer	Operation, Anti-tumor drug	no change
8	2010	de Seigneux [22]	62	F	IgGλ	IgG1λ	subepi, subendo	MGUS	Dexamethasone, Thalidomide	remission
9			n/a	n/a	no	IgG1κ	n/a	CLL	CY, Fludarabine	remission
10			n/a	n/a	no	IgG1κ	n/a	Unknown	PSL	ESRD
11	2011	Guiard [23]	n/a	n/a	IgGλ	IgG1λ	n/a	MM	PSL, Melphalan	remission
12			n/a	n/a	no	IgG2κ	n/a	Unknown	Rituximab	remission
13			n/a	n/a	no	IgG3κ	n/a	Unknown	PSL, CY	remission
14			n/a	n/a	no	IgG3κ	n/a	Unknown	Rituximab	decreased proteinuria
15	2012	Yamada [24]	63	M	no	IgG1λ	subepi	HCV	PSL, Mizoribine	decreased proteinuria
16	2012	Ito [25]	68	F	no	IgG3λ	subepi, mes	MPO-ANCA	PSL	remission
17	2013	Ohashi [26]	27	F	no	IgG2κ	subepi, mes	Idiopathic	PSL	remission
18	2014	Omokawa [16]	62	F	no	IgG4κ	subepi, mes	IgG4RD(lung)	mPSL, PSL	decreased proteinuria
19	2017	Present case	65	M	no	IgA1λ	subepi	IgG4RKD(TIN)	PSL	no change

Abbreviations: *CY* cyclophosphamide, *EM* electron microscopic study, *HCV* hepatitis C virus infection, *IEP* immunoelectrophoresis, *IF* immunofluorescent study, *IgG4RD* IgG4 related disease, *IgG4RKD* IgG4 related kidney disease, *mes* mesangial area, *MGUS* monoclonal gammopathy of undetermined significance, *ML* malignant lymphoma, *MM* multiple myeloma, *MPO-ANCA* myeloperoxidase-anti-neutrophil cytoplasmic antibody, *mPSL* methylprednisolone, n/a: not available, *PSL* prednisolone, *subendo* subendothelial area, *subepi* subepithelial area, *TIN* tubulointerstitialnephritis

Only one case of IgA1-λ-type monoclonal deposition with membranous features, which was associated with hepatitis C virus (HCV) infection and rectal cancer, has been reported [15]. The electron-dense deposits in the GBM were also irregular and similar to our case. They were also shaped like snow leopard spots. However, our patient was HCV negative and had no malignancy in the past 4 years.

Another relevant interesting case was IgG4-RD associated with non-Randall MIDD with membranous features [16]. It was reported as IgG4-related lung disease associated with IgG4-κ-type MIDD with membranous features but without tubulointerstitial change in the kidney. Steroid therapy was effective for both kidney and lung lesions. Thus, the authors mentioned possible common mechanisms in the formation of both IgG4-κ-type MIDD with membranous features and IgG4-RD.

In IgG4-RD, the markedly high IgG4 levels may simply be a reflection of the response to some primary inflammatory stimuli [17]. T cells have recently been suggested to contribute to the pathogenesis of IgG4-RD. Some reports have indicated that the production of T helper (Th) 2 cytokines and regulatory T-cell cytokines is increased in patients with IgG4-RD. A recent study showed that the Th17 cell subset is also upregulated in patients with IgG4-RD [18]. These cytokines are suspected to be important in the pathogenesis of IgG4-RD.

In PGNMID, the pathogenesis remains elusive. Because up to two-thirds of patients with PGNMID have no detectable M protein even after a long follow-up, Nasr et al. proposed that this unique glomerulonephritis may arise in the course of normal immune responses in these patients. During an immune response, 1 or more clones of B cells may proliferate and produce monoclonal IgG molecules with the ability to self-aggregate and rapidly deposit in the glomeruli [19].

In the present case, paraprotein in blood and urine was not detected by immunoelectrophoresis, and even in serum FLC assay, no deviation of the FLC ratio could be found. Therefore, 1 or more clones of B cells may have proliferated and produced monoclonal IgA molecules similarly to PGNMID.

Indeed, it is difficult to find a common mechanism between the 2 rare diseases, and they would not have a specific relationship.

IgG4-RD cause disorders of the basis of the immune response such as regulatory T-cell, and tend to cause other autoimmune diseases such as membranous nephropathy. However, it is unknown whether monoclonal immunoglobulin deposition increases as in the present case.

In summary, non-Randall MIDD with membranous features is rather rare, and only 1 case of monoclonal deposition from the IgA variant has been reported. To our knowledge, this is the first reported case of IgA1-lambda-type non-Randall monoclonal immunoglobulin deposition disease associated with membranous features in a patient with IgG4-related TIN.

## Abbreviations

CT: Computed tomography
GBM: Glomerular basement membrane
HCV: Hepatitis C virus
IgG4-RD: IgG4-related disease
MIDD: Monoclonal immunoglobulin deposition disease
MN: Membranous nephropathy
PGNMID: Proliferative glomerulonephritis with monoclonal IgG deposits
TIN: Tubulointerstitial nephritis
